# 3-(1*H*-Imidazo[4,5-*f*][1,10]phenanthrolin-2-yl)benzonitrile methanol solvate

**DOI:** 10.1107/S1600536809051472

**Published:** 2009-12-04

**Authors:** Wen-Lan Wang

**Affiliations:** a College of Chemistry and Biology, Beihua University, Jilin City 132013, People’s Republic of China

## Abstract

In the title compound, C_20_H_11_N_5_·CH_3_OH, the benzene ring is twisted by a small dihedral angle of 1.89 (11)° with respect to the imidazo[4,5-*f*][1,10]phenanthroline ring system. N—H⋯O and O—H⋯N hydrogen bonding is present in the crystal structure.

## Related literature

For related structures, see: Sun *et al.* (2007 ▶); Yin (2008 ▶); Zhang *et al.* (2008 ▶).
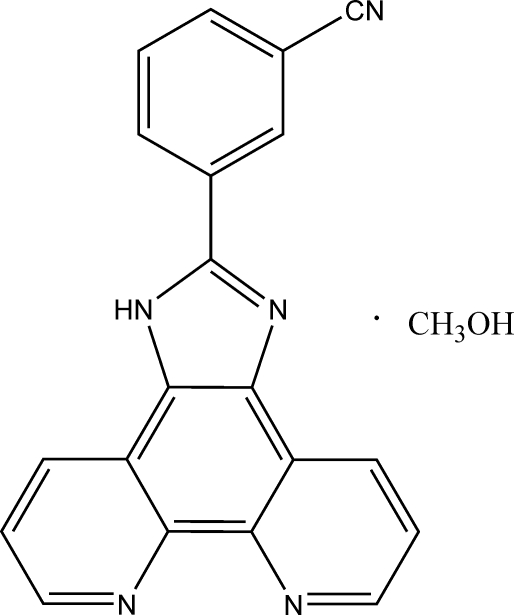


## Experimental

### 

#### Crystal data

C_20_H_11_N_5_·CH_4_O
*M* *_r_* = 353.38
Monoclinic, 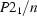

*a* = 7.115 (1) Å
*b* = 18.385 (2) Å
*c* = 13.5576 (12) Å
β = 99.956 (19)°
*V* = 1746.7 (4) Å^3^
*Z* = 4
Mo *K*α radiation
μ = 0.09 mm^−1^
*T* = 293 K
0.30 × 0.28 × 0.26 mm

#### Data collection

Rigaku, SCXmini diffractometer
15971 measured reflections
3432 independent reflections
2018 reflections with *I* > 2σ(*I*)
*R*_int_ = 0.099

#### Refinement

*R*[*F* ^2^ > 2σ(*F* ^2^)] = 0.072
*wR*(*F* ^2^) = 0.179
*S* = 1.04
3432 reflections
245 parameters
H-atom parameters constrained
Δρ_max_ = 0.18 e Å^−3^
Δρ_min_ = −0.19 e Å^−3^

### 

Data collection: *CrystalClear* (Rigaku, 2005 ▶); cell refinement: *CrystalClear*; data reduction: *CrystalClear*; program(s) used to solve structure: *SHELXS97* (Sheldrick, 2008 ▶); program(s) used to refine structure: *SHELXL97* (Sheldrick, 2008 ▶); molecular graphics: *ORTEP-3 for Windows* (Farrugia, 1997 ▶); software used to prepare material for publication: *SHELXL97*.

## Supplementary Material

Crystal structure: contains datablocks I, global. DOI: 10.1107/S1600536809051472/xu2700sup1.cif

Structure factors: contains datablocks I. DOI: 10.1107/S1600536809051472/xu2700Isup2.hkl

Additional supplementary materials:  crystallographic information; 3D view; checkCIF report

## Figures and Tables

**Table 1:** Hydrogen-bond geometry (Å, °)

*D*—H⋯*A*	*D*—H	H⋯*A*	*D*⋯*A*	*D*—H⋯*A*
N3—H3*B*⋯O1	0.86	1.95	2.803 (3)	174
O1—H1*E*⋯N5^i^	0.98	1.89	2.857 (3)	168

Symmetry code: (i) 
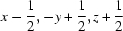
.

**Fig. 1.**
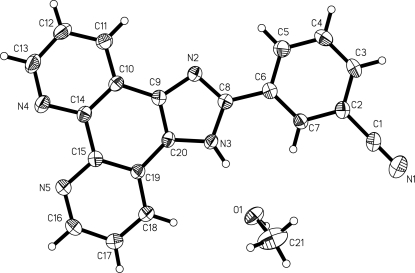
The asymmetric unit of the title compound with atom labels. Displacement ellipsoids were drawn at the 30% probability level.

**Fig. 2.**
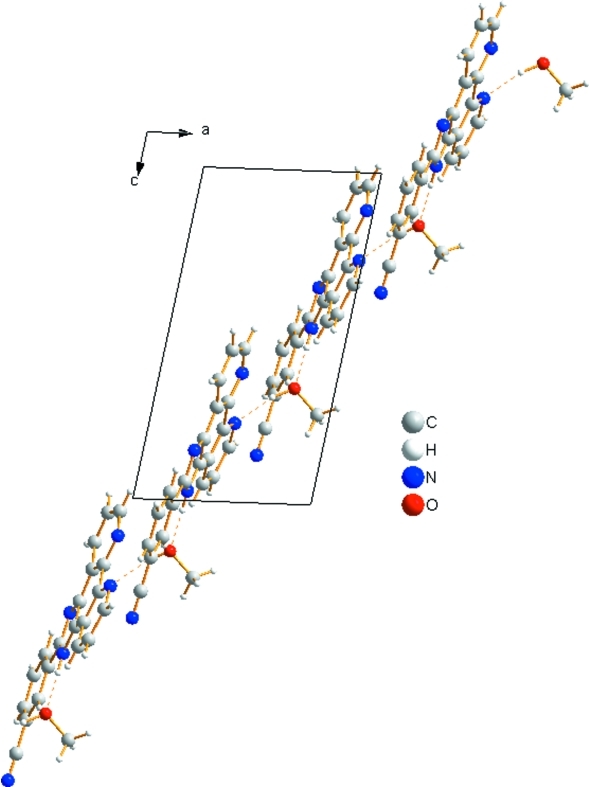
The packing viewed along the b axis. Hydrogen bonds are drawn as dashed lines

## supplementary crystallographic information

### Comment

1,10-Phenanthroline and its derivatives are commonly used as ligands in metal-organic coordination polymers (Sun *et al.*, 2007; Yin, 2008; Zhang *et al.*, 2008). The title compound was synthesized from 1,10-phenanthroline-5,6-dione.

The asymmetric unit of the title compound, C_20_H_11_N_5_.CH_3_OH, contains one organic molecule and one solvent methanol molecule (Fig.1). The molecules are connected by N—H···O and O—H···N hydrogen bonding to form a one-dimensional chain (Fig. 2). The organic molecule is essentially planar.

### Experimental

1,10-Phenanthroline-5,6-dione (1.5 mmol) and 3-cyanobenzaldehyde (1.5 mmol) were dissolved in CH_3_COOH and CH_3_COONH_4_ (1:1) solution (30 ml). The mixture was refluxed for 1.5 h under argon, after cooling this mixture was diluted with water and neutralized with concentrated aqueous ammonia, immediately resulting a yellow precipitate, which was washed with water, acetone and diethyl ether respectively. Crystals of the title compound were obtained by recrystallization from dichloromethane-methanol solution.

### Refinement

Methanol H atom was located in a difference Fourier map and refined as riding in as-found relative position, the thermal parameter was refined. Other H atoms were placed in calculated positions with C—H = 0.93 Å (aromatic), 0.96 Å (methyl) and N—H = 0.86 Å, and refined using a riding model, with *U*_iso_(H) = 1.5*U*_eq_(C) for methyl H  and 1.2*U*_eq_(C,N) for the others.

### Crystal data

**Table:**

C_20_H_11_N_5_·CH_4_O	*F*(000) = 736
*M**_r_* = 353.38	*D*_x_ = 1.344 Mg m^−^^3^
Monoclinic, *P*2_1_/*n*	Mo *K*α radiation, λ = 0.71073 Å
Hall symbol: -P 2yn	Cell parameters from 2018 reflections
*a* = 7.115 (1) Å	θ = 3.0–26.0°
*b* = 18.385 (2) Å	µ = 0.09 mm^−^^1^
*c* = 13.5576 (12) Å	*T* = 293 K
β = 99.956 (19)°	Block, colorless
*V* = 1746.7 (4) Å^3^	0.30 × 0.28 × 0.26 mm
*Z* = 4	

### Data collection

**Table:**

Rigaku, SCXmini diffractometer	2018 reflections with *I* > 2σ(*I*)
Radiation source: fine-focus sealed tube	*R*_int_ = 0.099
graphite	θ_max_ = 26.0°, θ_min_ = 3.0°
Detector resolution: 13.6612 pixels mm^-1^	*h* = −8→8
ω scan	*k* = −22→22
15971 measured reflections	*l* = −16→16
3432 independent reflections	

### Refinement

**Table:**

Refinement on *F*^2^	Primary atom site location: structure-invariant direct methods
Least-squares matrix: full	Secondary atom site location: difference Fourier map
*R*[*F*^2^ > 2σ(*F*^2^)] = 0.072	Hydrogen site location: inferred from neighbouring sites
*wR*(*F*^2^) = 0.179	H-atom parameters constrained
*S* = 1.04	*w* = 1/[σ^2^(*F*_o_^2^) + (0.0784*P*)^2^ + 0.0647*P*] where *P* = (*F*_o_^2^ + 2*F*_c_^2^)/3
3432 reflections	(Δ/σ)_max_ < 0.001
245 parameters	Δρ_max_ = 0.18 e Å^−^^3^
0 restraints	Δρ_min_ = −0.19 e Å^−^^3^

### Special details

**Table:**

Geometry. All e.s.d.'s (except the e.s.d. in the dihedral angle between two l.s. planes) are estimated using the full covariance matrix. The cell e.s.d.'s are taken into account individually in the estimation of e.s.d.'s in distances, angles and torsion angles; correlations between e.s.d.'s in cell parameters are only used when they are defined by crystal symmetry. An approximate (isotropic) treatment of cell e.s.d.'s is used for estimating e.s.d.'s involving l.s. planes.
Refinement. Refinement of *F*^2^ against ALL reflections. The weighted *R*-factor *wR* and goodness of fit *S* are based on *F*^2^, conventional *R*-factors *R* are based on *F*, with *F* set to zero for negative *F*^2^. The threshold expression of *F*^2^ > σ(*F*^2^) is used only for calculating *R*-factors(gt) *etc*. and is not relevant to the choice of reflections for refinement. *R*-factors based on *F*^2^ are statistically about twice as large as those based on *F*, and *R*- factors based on ALL data will be even larger.

### Fractional atomic coordinates and isotropic or equivalent isotropic displacement parameters (Å^2^)

**Table:**

	*x*	*y*	*z*	*U*_iso_*/*U*_eq_	
C1	0.6541 (5)	−0.05805 (18)	0.7761 (3)	0.0518 (8)	
C2	0.6702 (4)	−0.07430 (16)	0.6737 (2)	0.0430 (7)	
C3	0.6441 (4)	−0.14493 (17)	0.6377 (3)	0.0510 (8)	
H3A	0.6173	−0.1822	0.6795	0.061*	
C4	0.6581 (5)	−0.15944 (17)	0.5396 (3)	0.0538 (9)	
H4A	0.6398	−0.2066	0.5152	0.065*	
C5	0.6993 (4)	−0.10430 (17)	0.4769 (2)	0.0501 (8)	
H5A	0.7079	−0.1149	0.4107	0.060*	
C6	0.7281 (4)	−0.03325 (15)	0.5117 (2)	0.0386 (7)	
C7	0.7123 (4)	−0.01864 (16)	0.6113 (2)	0.0407 (7)	
H7A	0.7300	0.0285	0.6360	0.049*	
C8	0.7695 (4)	0.02402 (15)	0.4435 (2)	0.0391 (7)	
C9	0.8262 (4)	0.08115 (15)	0.3144 (2)	0.0388 (7)	
C10	0.8600 (4)	0.10077 (16)	0.2162 (2)	0.0391 (7)	
C11	0.8394 (4)	0.05275 (17)	0.1346 (2)	0.0490 (8)	
H11A	0.7998	0.0051	0.1413	0.059*	
C12	0.8787 (5)	0.07721 (19)	0.0448 (2)	0.0567 (9)	
H12A	0.8629	0.0470	−0.0110	0.068*	
C13	0.9430 (5)	0.14836 (19)	0.0393 (2)	0.0596 (10)	
H13A	0.9758	0.1633	−0.0210	0.072*	
C14	0.9157 (4)	0.17313 (16)	0.2013 (2)	0.0411 (7)	
C15	0.9232 (4)	0.22708 (16)	0.2820 (2)	0.0407 (7)	
C16	0.9749 (5)	0.34573 (18)	0.3349 (3)	0.0622 (10)	
H16A	1.0103	0.3930	0.3221	0.075*	
C17	0.9213 (5)	0.33164 (18)	0.4271 (2)	0.0592 (9)	
H17A	0.9197	0.3687	0.4737	0.071*	
C18	0.8714 (5)	0.26248 (17)	0.4477 (2)	0.0511 (8)	
H18A	0.8365	0.2515	0.5090	0.061*	
C19	0.8732 (4)	0.20771 (15)	0.3752 (2)	0.0388 (7)	
C20	0.8320 (4)	0.13278 (15)	0.3885 (2)	0.0371 (7)	
C21	0.9291 (6)	0.1623 (3)	0.7300 (3)	0.1045 (16)	
H21A	0.9003	0.1877	0.7875	0.157*	
H21B	1.0353	0.1853	0.7075	0.157*	
H21C	0.9610	0.1127	0.7478	0.157*	
N1	0.6392 (5)	−0.04524 (19)	0.8570 (2)	0.0749 (9)	
N2	0.7870 (3)	0.01301 (12)	0.34899 (16)	0.0406 (6)	
N3	0.7950 (3)	0.09555 (12)	0.47126 (17)	0.0390 (6)	
H3B	0.7891	0.1137	0.5291	0.047*	
N4	0.9610 (4)	0.19635 (14)	0.11301 (19)	0.0550 (7)	
N5	0.9787 (4)	0.29620 (13)	0.26399 (19)	0.0513 (7)	
O1	0.7700 (4)	0.16399 (13)	0.65327 (16)	0.0640 (7)	
H1E	0.6578	0.1781	0.6823	0.14 (2)*	

### Atomic displacement parameters (Å^2^)

**Table:**

	*U*^11^	*U*^22^	*U*^33^	*U*^12^	*U*^13^	*U*^23^
C1	0.050 (2)	0.061 (2)	0.044 (2)	−0.0024 (16)	0.0072 (16)	0.0113 (16)
C2	0.0349 (16)	0.050 (2)	0.0432 (18)	0.0017 (13)	0.0034 (14)	0.0069 (14)
C3	0.051 (2)	0.0400 (19)	0.063 (2)	−0.0021 (14)	0.0118 (17)	0.0147 (16)
C4	0.055 (2)	0.0383 (19)	0.070 (2)	−0.0027 (14)	0.0155 (18)	−0.0017 (17)
C5	0.0488 (19)	0.050 (2)	0.052 (2)	−0.0018 (15)	0.0117 (16)	−0.0018 (16)
C6	0.0300 (16)	0.0410 (17)	0.0446 (17)	0.0024 (12)	0.0063 (13)	0.0044 (13)
C7	0.0409 (17)	0.0381 (17)	0.0426 (18)	−0.0026 (13)	0.0060 (14)	0.0031 (13)
C8	0.0345 (16)	0.0416 (17)	0.0414 (17)	0.0021 (12)	0.0074 (13)	0.0001 (13)
C9	0.0359 (16)	0.0419 (18)	0.0382 (16)	−0.0003 (12)	0.0051 (13)	0.0016 (13)
C10	0.0351 (16)	0.0460 (18)	0.0361 (16)	0.0024 (13)	0.0062 (13)	−0.0013 (13)
C11	0.053 (2)	0.0483 (19)	0.0457 (19)	0.0018 (15)	0.0095 (16)	−0.0034 (15)
C12	0.070 (2)	0.062 (2)	0.0386 (19)	0.0111 (17)	0.0105 (17)	−0.0064 (16)
C13	0.078 (3)	0.066 (2)	0.0375 (19)	0.0088 (19)	0.0183 (17)	0.0087 (17)
C14	0.0407 (17)	0.0470 (19)	0.0357 (16)	0.0054 (13)	0.0071 (13)	0.0038 (13)
C15	0.0378 (17)	0.0422 (18)	0.0422 (17)	0.0020 (13)	0.0077 (14)	0.0027 (13)
C16	0.087 (3)	0.0392 (19)	0.063 (2)	−0.0115 (17)	0.021 (2)	−0.0003 (17)
C17	0.079 (3)	0.049 (2)	0.052 (2)	−0.0060 (17)	0.0183 (18)	−0.0075 (16)
C18	0.064 (2)	0.049 (2)	0.0429 (18)	−0.0016 (16)	0.0161 (16)	−0.0037 (15)
C19	0.0389 (17)	0.0409 (18)	0.0377 (16)	0.0005 (12)	0.0099 (13)	−0.0005 (13)
C20	0.0347 (16)	0.0436 (18)	0.0345 (16)	0.0030 (12)	0.0103 (13)	0.0041 (13)
C21	0.082 (3)	0.134 (4)	0.092 (3)	0.011 (3)	−0.002 (3)	−0.042 (3)
N1	0.071 (2)	0.100 (3)	0.053 (2)	−0.0063 (18)	0.0090 (17)	0.0078 (18)
N2	0.0423 (15)	0.0424 (15)	0.0386 (14)	−0.0004 (11)	0.0114 (11)	0.0001 (11)
N3	0.0434 (14)	0.0430 (15)	0.0322 (13)	0.0006 (11)	0.0110 (11)	−0.0003 (11)
N4	0.073 (2)	0.0573 (18)	0.0366 (15)	0.0024 (14)	0.0161 (14)	0.0035 (13)
N5	0.0651 (18)	0.0425 (16)	0.0489 (16)	−0.0064 (13)	0.0171 (13)	−0.0016 (12)
O1	0.0695 (17)	0.0778 (17)	0.0474 (14)	0.0083 (13)	0.0177 (13)	−0.0119 (12)

### Geometric parameters (Å, °)

**Table:**

C1—N1	1.145 (4)	C12—H12A	0.9300
C1—C2	1.444 (4)	C13—N4	1.323 (4)
C2—C3	1.388 (4)	C13—H13A	0.9300
C2—C7	1.393 (4)	C14—N4	1.361 (4)
C3—C4	1.376 (4)	C14—C15	1.471 (4)
C3—H3A	0.9300	C15—N5	1.365 (4)
C4—C5	1.387 (4)	C15—C19	1.416 (4)
C4—H4A	0.9300	C16—N5	1.328 (4)
C5—C6	1.392 (4)	C16—C17	1.393 (4)
C5—H5A	0.9300	C16—H16A	0.9300
C6—C7	1.400 (4)	C17—C18	1.362 (4)
C6—C8	1.465 (4)	C17—H17A	0.9300
C7—H7A	0.9300	C18—C19	1.409 (4)
C8—N2	1.324 (3)	C18—H18A	0.9300
C8—N3	1.371 (3)	C19—C20	1.426 (4)
C9—C20	1.378 (4)	C20—N3	1.377 (3)
C9—N2	1.383 (3)	C21—O1	1.400 (4)
C9—C10	1.439 (4)	C21—H21A	0.9600
C10—C11	1.403 (4)	C21—H21B	0.9600
C10—C14	1.412 (4)	C21—H21C	0.9600
C11—C12	1.371 (4)	N3—H3B	0.8600
C11—H11A	0.9300	O1—H1E	0.9842
C12—C13	1.392 (5)		
			
N1—C1—C2	179.2 (4)	C12—C13—H13A	117.4
C3—C2—C7	120.2 (3)	N4—C14—C10	122.4 (3)
C3—C2—C1	120.2 (3)	N4—C14—C15	117.3 (3)
C7—C2—C1	119.5 (3)	C10—C14—C15	120.3 (3)
C4—C3—C2	119.6 (3)	N5—C15—C19	121.3 (3)
C4—C3—H3A	120.2	N5—C15—C14	118.0 (3)
C2—C3—H3A	120.2	C19—C15—C14	120.8 (3)
C3—C4—C5	120.6 (3)	N5—C16—C17	124.4 (3)
C3—C4—H4A	119.7	N5—C16—H16A	117.8
C5—C4—H4A	119.7	C17—C16—H16A	117.8
C4—C5—C6	120.8 (3)	C18—C17—C16	118.6 (3)
C4—C5—H5A	119.6	C18—C17—H17A	120.7
C6—C5—H5A	119.6	C16—C17—H17A	120.7
C5—C6—C7	118.4 (3)	C17—C18—C19	119.3 (3)
C5—C6—C8	119.6 (3)	C17—C18—H18A	120.3
C7—C6—C8	122.0 (3)	C19—C18—H18A	120.3
C2—C7—C6	120.4 (3)	C18—C19—C15	118.5 (3)
C2—C7—H7A	119.8	C18—C19—C20	125.1 (3)
C6—C7—H7A	119.8	C15—C19—C20	116.4 (3)
N2—C8—N3	112.4 (2)	N3—C20—C9	105.4 (2)
N2—C8—C6	124.3 (3)	N3—C20—C19	131.0 (3)
N3—C8—C6	123.2 (3)	C9—C20—C19	123.6 (3)
C20—C9—N2	111.0 (3)	O1—C21—H21A	109.5
C20—C9—C10	120.9 (3)	O1—C21—H21B	109.5
N2—C9—C10	128.1 (3)	H21A—C21—H21B	109.5
C11—C10—C14	118.3 (3)	O1—C21—H21C	109.5
C11—C10—C9	124.1 (3)	H21A—C21—H21C	109.5
C14—C10—C9	117.6 (3)	H21B—C21—H21C	109.5
C12—C11—C10	119.0 (3)	C8—N2—C9	104.4 (2)
C12—C11—H11A	120.5	C8—N3—C20	106.8 (2)
C10—C11—H11A	120.5	C8—N3—H3B	126.6
C11—C12—C13	118.4 (3)	C20—N3—H3B	126.6
C11—C12—H12A	120.8	C13—N4—C14	116.7 (3)
C13—C12—H12A	120.8	C16—N5—C15	117.8 (3)
N4—C13—C12	125.2 (3)	C21—O1—H1E	108.4
N4—C13—H13A	117.4		

### Hydrogen-bond geometry (Å, °)

**Table:**

*D*—H···*A*	*D*—H	H···*A*	*D*···*A*	*D*—H···*A*
N3—H3B···O1	0.86	1.95	2.803 (3)	174
O1—H1E···N5^i^	0.98	1.89	2.857 (3)	168

Symmetry codes: (i) *x*−1/2, −*y*+1/2, *z*+1/2.
